# Unraveling the *In Vitro* Anti-Advanced Glycation End-Product (Anti-AGE) Potential of Fermented Red Cabbage and Beetroot: Insights into Composition and Activities

**DOI:** 10.3390/foods13121791

**Published:** 2024-06-07

**Authors:** Małgorzata Starowicz, Natalia Płatosz, Natalia Bączek, Dorota Szawara-Nowak, Kristýna Šimková, Wiesław Wiczkowski

**Affiliations:** Department of Chemistry and Biodynamics of Food, Institute of Animal Reproduction and Food Research of Polish Academy of Sciences, 10 Tuwima Street, 10-748 Olsztyn, Poland; m.starowicz@pan.olsztyn.pl (M.S.); n.baczek@pan.olsztyn.pl (N.B.); d.szawara-nowak@pan.olsztyn.pl (D.S.-N.); simkova.kris@gmail.com (K.Š.); w.wiczkowski@pan.olsztyn.pl (W.W.)

**Keywords:** AGEs, red beetroot, red cabbage, glycation inhibitors, phytochemicals, technological processes

## Abstract

This study verified the *in vitro* activity of red cabbage and beetroot against the formation of advanced glycation end-products (AGEs) and their relationship with the biomolecules’ content. Fermentation of cabbage increased the total phenolic (~10%) and flavonoid contents (~14%), whereas decreased total phenolics/flavonoids in beetroot. Fermented cabbage exhibited higher ability against AGEs, i.e., 17% in the bovine serum albumin–methylglyoxal (BSA-MGO) model and 25% in the BSA–glucose model, while beetroot exhibited 23% and 18%, respectively. The major compounds of cabbage products were cyanidin 3-(sinapoyl)(sinapoyl)-diglucoside-5-glucoside, sinapic acid, and epicatechin. Syringic acid and epicatechin were predominantly present in fermented beetroot. 2,17-bidecarboxy- and 2,15,17-tridecarboxy-betanin were the major betalains. Fermented vegetables can be effective inhibitors of the AGE formation/accumulation and could be recommended in the prevention of diet-related diseases.

## 1. Introduction

After fruits, vegetables are colorful and flavorful natural food products with a high content of various biologically active compounds, e.g., carotenoids, glucosinolates, and phenolics [1]. Red cabbage (*Brassica oleracea* L.) and red beetroot (*Beta vulgaris*) are examples of vegetables widely used for nutritional purposes, primarily due to their antioxidant, anti-diabetic, anti-cancer, anti-hyperglycemic, cardioprotective, and hypolipidemic properties [2]. The documented health benefits of these vegetables, which are the so-called functional foods, are strongly linked to the high amount of their bioactive molecules. A high content of pigments, like anthocyanins, was found in red cabbage, whereas betalains were shown to be functionally the most important phytochemicals of beetroot [3,4].

In the context of food processing, one interesting technique to further enhance the bioavailability and health benefits of these bioactive compounds is fermentation. Fermentation is a natural process that involves the controlled growth of microorganisms like lactic acid bacteria (LAB) on vegetables. As a part of this process, the bioactive compounds present in red cabbage and red beetroot can undergo various transformations. Therefore, by subjecting red cabbage and red beetroot to the fermentation process, we can potentially enhance the nutritional benefits of these vegetables. Fermentation may lead to increased bioavailability of anthocyanins and betalains, resulting in a fermented product that may offer not only the original nutritional advantages but also additional health-promoting properties associated with the activity of the beneficial microorganisms.

It has already been shown that the extracts of some edible plants serve as natural inhibitors of advanced glycation end-product (AGE) formation, e.g., selected fruits, mung bean, buckwheat hull and seeds, teas, spices, and herbs [5,6]. AGEs are harmful compounds that result from the non-enzymatic reaction between sugars and proteins or lipids in our bodies. AGE accumulation *in vivo* has been implicated to be the major pathogenic process in diabetic complications, like atherosclerosis, Alzheimer’s disease, and aging. There are several effective inhibitors of AGEs, like aminoguanidine and carnosine (direct AGE-lowering compounds) or aspirin and metformin (indirect therapy) [7]. Because of the side effects of AGE inhibitor usage, natural inhibitors were addressed in several investigations [6,8,9]. Studies have demonstrated that beetroot and red cabbage exhibit potent anti-glycation activity; however, they failed to find a correction between the phytochemicals and the obtained anti-glycation activity [10,11]. To the best of our knowledge, there are no data describing the effect of the bioactive compounds contained in red cabbage and red beetroot on the potential benefits of fermentation, which may make fermented versions of these vegetables even more desirable as functional foods supporting general health. This fermentation process may enhance the availability and bioactivity of the beneficial compounds, making them even more effective as inhibitors of AGE formation.

Taking the above-mentioned literature findings into account, this study aimed to compare the ability to inhibit AGE formation between fresh and fermented vegetables and define bioactive compounds that are responsible for their anti-glycation properties. To achieve the goal, in this study, the following parameters were determined: (1) profile and content of anthocyanins, betalains, phenolic acids, and flavonoids; (2) anti-glycation *in vitro* study using two models, bovine serum albumin and methylglyoxal (BSA-MGO) and BSA with glucose (BSA–glucose); and (3) total phenolic and flavonoid contents in fresh and fermented products of red cabbage and red beetroot.

## 2. Materials and Methods

### 2.1. Chemicals

Aluminum chloride hexahydrate (≥99%), bovine serum albumin (BSA; ≥96%), hydrogen chloride (HCl), diethyl ether, D-glucose (≥96%), methylglyoxal (MGO), trifluoroacetic acid (TFA), sodium hydroxide (NaOH), and MS grade reagents, including acetonitrile, methanol (MeOH), water, and formic acid (FA), were purchased from Sigma-Aldrich (St. Louis, MO, USA). Folin’s phenol reagent, hydrochloric acid, and sodium hydroxide were obtained from POCH S.A. (Gliwice, Poland). Cyanidin chloride, phenolic acid (caffeic, chlorogenic, *p*-coumaric, gallic acid (GA), ferulic, *p*-hydroxybenzoic, *m*-hydroxyphenylacetic, *m*-hydroxybenzoic, isoferulic, protocatechuic, sinapic, syringic, and vanillic acids) and flavonoid (apigenin, epicatechin, kaempferol, quercetin (Q), orientin, rutin, and vitexin) standard compounds were obtained from Extrasynthese (Genay, Rodan, France) and Sigma Chemical Co. (St. Louis, MO, USA). In addition, several anthocyanin standards were purchased according to a previous publication [12].

### 2.2. Preparation of Samples

Red cabbage (*Brassica oleracea* L. var. *capitata*) and red beetroot (*Beta vulgaris* L. subsp. *vulgaris*) were purchased at a local market (Olsztyn, Poland). The fermentation process was carried out as described by Płatosz et al. [12]. Briefly, the plant materials (1.5 kg) were thoroughly cleaned and chopped into thick slices (2–3 mm). Then, the prepared materials were placed in sterilized stoneware dishes and immersed along with 12 g of salt, 12 g of sugar, and 1.5 L of water. The spontaneous fermentation process was carried out separately for red cabbage and red beetroot for about 14 days in the dark and at room temperature (23 °C). Finally, the fermented samples (200 g) were freeze-dried, powdered, and frozen at −80 °C before further analysis.

### 2.3. Anti-Glycation Activity Measurement

The glycation inhibitory activity was evaluated *in vitro* as the ability to inhibit the formation of bovine serum albumin and glucose (BSA–glucose) and bovine serum albumin and methylglyoxal (BSA-MGO) adducts according to the previously proposed methodology of Starowicz and Zieliński (2019) [6]. Powdered samples (300 g) were extracted with 1 mL of 80% MeOH, shaken for 2 h at 25 °C, and centrifuged (16,000× *g* for 10 min). The BSA–glucose and BSA-MGO assays were conducted using a microplate reader (Infinite PRO 1000, Tecan, Männedorf, Switzerland) in a fluorescence mode set at λ = 360 and λ = 420 nm and λ = 340 and λ = 420 nm, respectively.

### 2.4. Measurement of Total Phenolic (TP) and Total Flavonoid (TF) Contents of Red Cabbage and Red Beetroot and Their Products

The TP content was determined according to Przygodzka et al. [13] at 725 nm after the addition of Folin’s phenol reagent to 80% MeOH extracts. The TF content was determined at 415 nm using aluminum chloride hexahydrate as the main reagent [13]. A microplate reader (Infinite PRO 1000, Tecan, Switzerland) was used in both methods. The calibration curve of TP was plotted based on the standard of GA (R^2^ = 0.998), whereas that of TF—Q as a reference (R^2^ = 0.986).

### 2.5. Determination of Contents of Phenolic Acids and Flavonoids by Liquid Chromatography in Red Cabbage and Red Beetroot and Their Products

The profile and content of phenolic acids and flavonoids were analyzed according to the method developed by Płatosz et al. [12]. About 0.15 g of the powder of red beetroot and red cabbage (fresh and fermented) samples were extracted with a mixture of methanol, water, and formic acid (80/19.9/0.1; *v*/*v*/*v*). The extraction process was iterated five times, resulting in crude extracts. Subsequently, free phenolic compounds were isolated from crude extracts using diethyl ether, following the adjustment of the extract to pH 2 with 6 M HCl. After obtaining the free forms, esters were hydrolyzed at room temperature with 4 M NaOH, and glycosides in the residues were hydrolyzed using 6 M HCl. The liberated free forms of phenolic acids and flavonoids were extracted with diethyl ether. The resulting ether extracts underwent evaporation to dryness under a stream of nitrogen, and the residue was reconstituted in 100 µL of 80% (*v*/*v*) methanol containing 0.95% (*v*/*v*) formic acid.

The high-performance liquid chromatography—HPLC system (LC-200, Eksigent, Vanghan, ON, Canada)—coupled with a mass spectrometer (QTRAP 5500, AB Sciex, Vaughan, ON, Canada) were utilized in this study and featured a HALO C18 column (2.7 μm particles, 0.5 × 50 mm, Eksigent, Vaughan, ON, Canada) maintained at 45 °C, with an eluent flow rate set at 15 μL/min. The eluent comprised a mixture of A (water/formic acid; 99.05/0.95; *v*/*v*) and B (acetonitrile/formic acid, 99.05/0.95, *v*/*v*). The gradient protocol was as follows: an initial 5% B for 0.1 min, a transition from 5% to 90% B within 1.9 min, a subsequent 90% B for 0.5 min, a shift from 90% to 5% B in 0.2 min, and finally, 5% B for 0.3 min. Qualitative and quantitative assessments were carried out based on the comparison of retention times (rt) and the presence of the respective parent and daughter ion pairs (Multiple Reaction Monitoring method, MRM) with data obtained after analysis of the authentic standards (Table S1). The external standards (0.01–0.5 µg/mL) had linear calibration curves with a coefficient of determination of 0.997–0.999. The results were expressed as µg/g dry matter (DM).

### 2.6. Determination of Anthocyanin Content in Fresh and Fermented Red Cabbage

Approximately 100 mg of the powdered samples were extracted 5 times with a mixture of MeOH/water/TFA (60/36/4, *v*/*v*/*v*) by sonication and vortexing according to the method previously reported by Wiczkowski et al. [3].

Chromatographic assessments were carried out using an HPLC system with diode array detection (DAD) at 520 nm (Shimadzu, Kyoto, Japan) at 45 °C, using a flow rate of 0.2 mL/min on a 150 × 2.1 mm i.d. XBridge C18 3.5 μm column (Waters, Milford, MA, USA). The elution of anthocyanins followed a gradient system consisting of water/formic acid (phase A: 99.4:0.6, *v*/*v*) and acetonitrile/formic acid (phase B: 99.4:0.6, *v*/*v*). The gradient sequence comprised 3–17% B (0–77 min), 17–80% B (77–80 min), 80–3% B (80–84 min), and 3% B (84–105 min). Compound identity was confirmed using a mass spectrometer (QTRAP 5500, AB SCIEX, Vaughan, ON, Canada) under specific conditions: curtain gas at 20 L/min, collision gas at 9 L/min, ion spray voltage at 5300 V, temperature at 550 °C, ion source gas 1 at 55 L/min, ion source gas 2 at 45 L/min, declustering potential from 60–180 V, entrance potential at 10 V, collision energy from 20 to 40 eV, collision cell exit potential at 10–15 V, and ionization in positive mode. Anthocyanins were identified through a comparative analysis of their retention time, UV–visible spectrum, and MS/MS fragmentation spectrum (mass-to-charge ratio; *m*/*z* values) against previously published data (Table S2). The quantification of anthocyanins was accomplished by calculating the HPLC–DAD peak area at 520 nm against cyanidin as the external standard. The calibration curve (the range of 0.01–0.5 μg/mL) was linear with R^2^ = 0.998.

### 2.7. Extraction and Chromatographic Analysis of Betalains in Fresh and Fermented Red Beetroot

Approximately 200 mg of dried and pulverized red beetroot samples underwent extraction through a vortex using the mixture of water/MeOH/TFA (84.95/15/0.05, *v*/*v*/*v*) and sonication using a VC 750 instrument (Sonics & Materials, Newtown, CT, USA) [11]. The resulting mixture was then centrifuged (Centrifuge 5415R, Eppendorf, Wesseling, Germany) for 10 min at 13,200× *g* and 4 °C. The supernatant was carefully collected in a 5 mL flask, and this entire process was repeated five times.

The assessment of extracts involved utilizing a micro-HPLC system (LC200, Eksigent, Vaughan, ON, Canada) connected with a TripleTOF 5600+ mass spectrometer (AB SCIEX, Vaughan, ON, Canada). Upon injecting 5 µL aliquots of sample solutions into the HPLC systems, compound separation occurred on a Gemini C18 3 µm 50 × 0.5 mm column (Phenomenex, Torrance, CA, USA) at 45 °C with a flow rate of 25 µL/min. Elution employed a solvent gradient system, comprising solvent A (0.012% formic acid aqueous solution with 5 mM ammonia) and solvent B (0.012% formic acid and 5% water acetonitrile solution with 5 mM ammonia). The gradient included 0% B (0–0.5 min), 0–90% B (0.5–2 min), 90% B (2–2.5 min), 90–0% B (2.5–2.7 min), and 0% B (2.7–3 min). Betalains were identified by comparing retention time, MS/MS fragmentation spectrum (*m*/*z* values), and λ_max_ values with published data or through the interpretation of the obtained fragmentation spectrum (Table S3). The quantities of betacyanins and betaxanthins were determined using micro-HPLC TOF peak area against betanin and vulgaxanthin I external standards. Calibration curves (0.01–0.5 μg/mL) were linear with correlation coefficients of 0.997 and 0.999. The results were expressed as µg/g dry matter (DM).

### 2.8. Statistical Analysis

The analyses were performed in triplicate, and the reported data are the mean results for each product with the standard deviation (Mean ± SD). The analysis of variance (ANOVA) followed by Tukey’s multiple comparison test were conducted. Differences between mean values were found to be significant at *p* < 0.05. The statistical study was implemented using Statistica 13.1 (Statsoft Inc., Tulsa, OH, USA).

## 3. Results and Discussion

### 3.1. Qualitative and Quantitative Identification of Anthocyanins in Products of Red Cabbage

The profile and content changes of anthocyanin compounds were determined in fresh red cabbage and after spontaneous 7-day fermentation (Table 1). Nineteen anthocyanins were identified in fresh red cabbage via micro-HPLC-MS/MS analysis, including two non-acylated and seventeen derivatives acylated with caffeic, ferulic, *p*-coumaric, and sinapic acid cyanidin. Furthermore, the sum of anthocyanins in fresh red cabbage was at 12.81 ± 0.41 µg/g DM. In this respect, the two non-acylated compounds (compound no. 1 and 2) that were the minority accounted for 16.63% of the sum of anthocyanins. Particularly, the main non-acylated form of cyanidin 3-diglucoside-5-glucoside (Cy3diG5G, no. 1), which was also the basic structure of all anthocyanins found in red cabbage, accounted for 14.36% of the sum of anthocyanins (1.84 ± 0.18 µg/g DM). In addition, it was found that acylated anthocyanins consisting of 10 mono-acylated (compound nos. 3, 4, 6, 7, 9, 11, 13–16; 4.50 ± 0.15 µg/g DM) and 7 di-acylated (compound nos. 5, 8, 10, 12, 17–19; 6.18 ± 0.08 µg/g DM) cyanidin derivatives were the main group of anthocyanins found in fresh red cabbage (approximately 83.37% of the sum of anthocyanins). Cyanidin 3-(sinapoyl)(sinapoyl)-diglucoside-5-glucoside (Cy3(sin)(sin)diG5G, no.19) (22.17% of the sum of anthocyanins) was the predominant pigment in the anthocyanin group. Furthermore, the first seven major cyanidin derivatives accounted for almost 73.53% of the sum of anthocyanins. A similar profile and content of anthocyanins in fresh red cabbage were presented in the study by Płatosz et al. and Wiczkowski et al. [3,12].

The profile and content of anthocyanin compounds in red cabbage after spontaneous fermentation were significantly different from that determined in fresh cabbage. In particular, the study tentatively identified eighteen cyanidin derivatives in fermented red cabbage, including two non-acylated, ten mono-acylated, and six di-acylated anthocyanins. Contrary to fresh red cabbage, cyanidin 3-(caffeoyl)(*p*-coumaroyl)-diglucoside-5-glucoside (Cy3(caf)(*p*-cum)diG5G, no. 12) was not identified in fermented red cabbage. Moreover, the sum of anthocyanins of fermented red cabbage (11.81 ± 0.34 µg/g DM) was significantly lower (by 7.8%) than that of the fresh one (12.81 ± 0.41 mg/g; *p* < 0.05). This observation is in agreement with that of a previous report by Wiczkowski et al. [3]. Ultimately, the analysis of fermentation effect on the percentage content of anthocyanin compounds in red cabbage indicated they can be ranked in the following descending order: di-acylated > mono-acylated > non-acylated. Although di-acylated anthocyanins had the highest content of anthocyanins, the fermentation process reduced them by 8.73%. The opposite phenomenon was observed in the case of mono- and non-acylated anthocyanins—the fermentation process increased their content by 8.71% and 6.9%, respectively. In the case of individual components from the group of anthocyanins, fermentation increased the content of four anthocyanins (nos. 1, 11, 13, 14), and the changes in content were statistically significant for three compounds (nos. 1, 11, 13, 14). In the case of the remaining fourteen anthocyanins (nos. 2–10, 13, 15–19), the fermentation process decreased their content; however, the decrease was statistically significant only in the case of ten of them (compound nos. 2, 5–9, 15, 17–19). Cy3(sin)(sin)diG5G (no. 19) and Cy3diG5G (no. 1) (22.10 and 16.17% of the sum of anthocyanins, respectively) were predominant compounds in the group of anthocyanins. As with fresh red cabbage, the first seven major cyanidin derivatives accounted for almost 76.81% of the sum of anthocyanins.

It is generally assumed that acylated anthocyanins show a higher thermal and/or chemical stability under different physicochemical conditions than the non-acylated compounds [14]. In addition, previous studies have suggested that anthocyanins acylated with sinapic acid have the lowest half-life and therefore are less stable compared to the anthocyanins acylated with ferulic and *p*-coumaric acids [15,16]. Also, the report by Sadilova et al. showed that the degradation of acylated anthocyanins led to the formation of acyl-glycosides and the corresponding aglycones [15]. As mentioned earlier, Cy3diG5G (no. 1) is the basic structure of all anthocyanins found in red cabbage; therefore, di-acylated cyanidin derivatives can convert to mono-acylated and then to non-acylated forms. These findings remain in agreement with our observations that were made to explain a decrease in di-acylated and an increase in mono-acylated and non-acylated anthocyanin contents after the fermentation process. Moreover, it should be emphasized that the stability of anthocyanins, and thus their profile and content in the final product, also depends on other external factors, such as the length and temperature of the technological process, the presence of light and oxygen, the activity of microorganisms and plant enzymes, and the pH value [17]. Therefore, it is very important to consciously select the parameters of technological processes in order to eliminate their negative effects on anthocyanin compounds, which in turn will contribute to increasing the health-promoting effects of these compounds in the final product.

### 3.2. Profile and Content of Betalains in Red Beetroot

Table 2 shows the profile and content of betalains in red beetroot (fresh and fermented). Betacyanins (red/violet-colored) and betaxanthins (yellow-colored pigments) are the representatives of betalains in red beetroot. Five compounds from the betacyanin group were detected in the present study, i.e., betanin, isobetanin, 17-decarboxy-, 2,17-bidecarboxy-, and 2,15,17-tridecarboxy-betanin. Moreover, two betaxanthins were identified in the analyzed samples: vulgaxantin I (glutamine–betaxanthin) and vulgaxantin II (glutamic acid–betaxanthin). The spectrum of betalains was not as wide as that presented by Sawicki et al. [11]; however, the most significant betalains, i.e., betain, isobetanin, vulgaxanthin I, and vulgaxanthin II, were determined [18].

As presented in Table 2, the total content of betalains was found within the range of 0.94–5.75 mg/g DM, which corresponds to the betalain content in flesh determined by Slatnar et al. and Paciulli et al. [18,19]. In fresh beetroot, the highest content of betanin was noticed (44% of the sum of betalains), followed by 2,5,17-tridecarboxybetanin (19%), isobetanin (13%), 2,17-dicarboxybetanin (12%), and vulgaxanthin II (8%). A different percentage share was noted in fermented beetroot. 2,17-Dicarboxybetanin was the major betalain determined after beetroot fermentation (36%). Vulgaxanthin II and 2,5,17-tridecarboxybetanin had also a high contribution in the betalain content, i.e., 27% and 24%, respectively. Furthermore, a very low content of betanin was determined, whereas isobetanin was not detected in the fermented product. Our study results are in agreement with the findings reported by Sawicki et al. [11], who observed the conversion of betanin into other forms after its exposure to increased temperature. The transformation is feasible due to the thermal treatment of red beetroot. The mechanism of betalain heat-induced transformation was recently investigated in detail by Sutor-Świeży et al. [20]. They observed a sequence of decarboxylation and further dehydrogenation of betanin/isobetanin (main betalains). The same results were recorded by Gokhale and Lele [21], who observed that rising drying temperature decreased the content of betacyanins and, on the other hand, increased the content of betaxanthins. The betacyanin content could also be influenced by long-time storage, whereas betaxanthins seem to be more stable molecules [21]. Fermentation caused a six-fold decrease in the total content of betalains. Among the factors enhancing betalain stability, 3 < pH < 7 are the proper conditions to avoid their degradation [22]. Moreover, the varied distribution of betalains was noted among the beetroot products. Fresh beetroot contained 89% of betacyanins and 11% of betaxanthins, while 69% of betacyanins and 31% of betaxanthins were detected in fermented beetroot. We identified a higher ratio of betacyanins to betaxanthins than that previously presented by Slatnar et al. [18]. This could be related to the likely differences in betalains among beetroot varieties. Each variety could have its unique betalain fingerprint. Moreover, the ratio between violet pigments and yellow ones was established at 1:0.12 in fresh beetroot and at 1:0.45 in fermented beetroot. Our study results are more related to those achieved by Nemzer et al. [23]. The availability of betalains is important due to their various health-promoting properties, including their anti-bacterial, anti-fungal, anti-cancer, anti-lipidemic, hepatoprotective, neuroprotective, and cardiovascular effects [22].

### 3.3. Total Phenolic and Flavonoid Contents (TPC and TFC)

The results of total phenolic content (TPC) and total flavonoid content (TFC) determined in red cabbage and red beetroot are presented in Table 3 and Table 4, respectively.

In red cabbage (Table 3), TPC was established to be 36.49 ± 1.25 mg GA/g DM, whereas TFC was to be 0.72 ± 0.03 mg Q/g DM. The fermentation process significantly increased these values by ~9% for TPC and 12% for TFC. The content of bioactive molecules varies depending on the vegetables’ variety and their harvest period. This could be a reason why TP contents determined by Oancea et al. and Tabart et al. were two times lower in comparison to our results [24,25].

In the case of red beetroot (Table 4), the TPC was 33.73 ± 0.79 mg GA/g DM, and the TFC was 2.11 ± 0.03 mg Q/g DM. About 22% and 33% decrease was noticed in TPC after beetroot fermentation. The same observation was made for beetroot’s TFC. The values of TFC dropped by about 31% after fermentation. Our findings are in agreement with Şengül et al., who noticed that the content of TP was higher in red cabbage than in beetroot [26]. The TPC and TFC measurements can be used to present correlations between samples. However, it should be taken into account that the Folin–Ciocalteu’s method may be susceptible to interference from non-phenolic compounds that may also react with the Folin–Ciocalteu’s reagent and thus lead to the overestimation of the phenolic content. The strongly reacting substances, other than phenols, include tertiary aliphatic amines, tertiary amine-containing biological buffers, tryptophan, hydroxylamine, hydrazine, certain purines, and other miscellaneous organic and inorganic reducing agents [27]. It is, therefore, recommended to determine the profile and content of phenolic compounds in selected materials using the chromatographic methodology with standards of chemicals. Therefore, in the next steps of the present study, non-anthocyanin phenolic compounds, including phenolic acids and flavonoids, anthocyanins, and betalains, were determined qualitatively and quantitatively in fresh red cabbage and red beetroot and their processed products.

### 3.4. Qualitative and Quantitative Identification of Non-Anthocyanin Phenolic Compounds (Phenolic Acids and Flavonoids) in Products of Red Cabbage and Red Beetroot

The effect of fermentation on the individual phenolic acids and flavonoids of red cabbage (Table 3) and red beetroot (Table 4) was studied. In red cabbage, we determined nineteen non-anthocyanin phenolic compounds that belong to two groups: phenolic acids and flavonoids (Table 3). Our study demonstrated that the sum of phenolic acids and flavonoids (SPF) in fresh red cabbage was 325.91 ± 14.01 µg/g DM. The phenolic compounds in fresh red cabbage were represented majorly by phenolic acids (on average, 92.55% of the SPF, 301.64 ± 13.37 µg/g DM), and flavonoids were a minority at the level of 7.45% (24.27 ± 0.64 µg/g DM) of the SPF. For comparison, the mean ratio of phenolic acids to flavonoids in red cabbage determined by Drozdowska et al. was 1:1 [28]. Such a large discrepancy in the proportion of compounds may be due to species, variety, degree of vegetable ripeness, harvest period, and storage conditions. The use of different extraction methodology can also cause significant differences between the compared results.

It is interesting that the content of phenolics significantly varied in the fresh and fermented red cabbage (*p* < 0.05) (Table 3). The spontaneous fermentation process greatly increased the sum of the non-anthocyanin phenolic compounds by approximately 21.56% of SPF, 396.17 ± 15.75 µg/g DM, compared to fresh red cabbage. In detail, the sum of phenolic acids (SP) increased significantly by 27.95% (385.95 ± 14.88 µg/g DM), while that of flavonoids (SF) decreased significantly, i.e., 42.11% (10.22 ± 0.87 µg/g DM). The observed changes are consistent with the previous work of Hunaefi et al., who showed that the fermentation process of red cabbage lasting up to 7 days led to an increase in the content of phenolics compared to unfermented red cabbage [29]. Furthermore, the one-week fermentation could result in a decrease in the content of phenolic compounds. The longer the fermentation time, the greater the reduction that could be noticed, i.e., even 4.5-fold on the 35th day of fermentation [29].

Three groups could be distinguished among the twelve phenolic acids detected in red cabbage: hydroxycinnamic acids (caffeic, chlorogenic, *p*-coumaric, ferulic, isoferulic, and sinapic acid), hydroxybenzoic acids (*p*-hydroxybenzoic, *m*-hydroxybenzoic, protocatechuic, syringic, and vanillic acid), and phenylacetic acid (*m*-hydroxyphenylacetic acid). As we mentioned earlier, red cabbage had a high content of phenolic acids. In detail, hydroxycinnamic acids accounted for 67.07% (202.30 ± 13.29 µg/g DM) of SP, followed by hydroxybenzoic acids (87.32 ± 2.05 µg/g DM, 28.95% of SP) and phenylacetic acid (12.02 ± 0.41 µg/g DM, 3.99% of SP). Similar results were reported by Drozdowska et al. for fresh red cabbage [28]. Furthermore, phenolic acids were present in the fresh red cabbage mainly in the bound forms (80.89% of the SPF), while free forms were in the minority (19.11% of SPF). The conducted analysis showed that the spontaneous seven-day fermentation positively affected the contents of phenolic acids in red cabbage products. In particular, it significantly increased the content of hydroxycinnamic acids (274.18 ± 10.09 µg/g DM, 71.04% of SP), while reducing the content of hydroxybenzoic acids and phenylacetic acid (25.17% and 3.79% of SP, respectively). In the case of free and conjugated forms of phenolic acids, the fermentation process simultaneously increased the total content of free and bound forms of phenolic acids by 57.88% and 20.88% of SPF, respectively, compared to fresh red cabbage. The higher content of phenolic acids in fermented red cabbage can be attributed to the effect of lactic acid bacteria strains. During the fermentation process, bacteria use soluble fiber for their growth, as a result of which phenolic compounds are released from the polymer structures of the fiber [30]. Of the twelve phenolic acids identified in the fresh and fermented red cabbage extracts, sinapic acid was the dominant compound (24.06% and 33.51% of SPF, respectively) followed by caffeic acid (14.81% and 15.81% of SPF, respectively) and syringic acid (12.16% and 11.18% of SPF, respectively). Our study results were in agreement with the available data indicating that the major phenolic acid in the extracts of fresh and fermented red cabbage was sinapic acid [28,29,31] or its derivatives [10,32]. Sinapic acid and its derivatives have been proven to elicit health-promoting effects, i.e., anti-bacterial, anti-cancer, anti-glycemic, anti-inflammatory, anti-mutagenic, antioxidant, and neuroprotective ones [33].

The performed analysis revealed that red cabbage and its fermented products were the notable sources of flavonoids. Widely distributed in the plant kingdom, flavonoids represent a class of phenolic compounds that show a wide diversity of biological activities observed *in vitro*, having a beneficial effect on the body [34]. The following seven flavonoids were detected in the fresh and fermented red cabbage: flavones (apigenin, orientin, vitexin), flavonols (rutin, kaempferol, quercetin), and flavanols (epicatechin) (Table 3). Drozdowska et al. (2020) determined nine flavonoid compounds in red cabbage products [28]. As shown in Table 3, the sum of flavonoids (SF) was 24.27 ± 0.64 µg/g DM, 7.45% of SPF. In particular, the content of flavanols in our study was 19.81 ± 0.45 µg/g DM, 6.08% of SPF. In turn, flavones and flavonols were in the minority and their contents averaged to be 3.36 ± 0.23 µg/g DM (1.03% of SPF) and 1.11 ± 0.06 µg/g DM (0.34% of SPF), respectively. At the same time, the analysis of the contents of free and conjugated flavonoids revealed that flavonoids were mainly in the conjugated forms (95.07% of the SPF; 23.09 ± 0.64 µg/g DM), and the free forms constituted only about 5% (1.20 ± 0.10 µg/g DM) of SPF. Epicatechin was found to be the major flavonoid found in fresh red cabbage (81.59% of the SF). The other flavonoids accounted for less than 1% of the SPF and could be ranked in the following descending order of content: orientin > apigenin > kaempferol > quercetin > rutin = vitexin. These findings differ from those available in the literature, indicating myricetin as the major compound of fresh red cabbage [28].

As with phenolic acids, the content of flavonoids in red cabbage was investigated as influenced by spontaneous fermentation. The fermentation processes significantly reduced the SF in red cabbage products by approximately 57.89% to 10.22 ± 0.87 µg/g DM (Table 3). Flavanols turned out to be major flavonoids of fermented red cabbage, and their content decreased in our study by about 72.79% (5.39 ± 0.45 µg/g DM). A similar trend was observed by us in the case of flavones. Their content decreased by about 18.15%, to the value of 2.75 ± 0.28 µg/g DM in the fermented product. On the other hand, the fermentation process increased the content of flavonols to 2.08 ± 0.14 µg/g DM (about 87.39%) in the final product. As in the case of fresh red cabbage, the flavonoids in the fermented product were mainly in the conjugated form (86.28%). Interestingly, the fermentation process increased the content of free forms of flavonoids by about 16.67%, while reducing the content of the conjugated forms by about 61.76%. According to our results, fermentation increased the contents of five compounds, i.e., kaempferol, orientin, quercetin, rutin, and vitexin, while the content of two compounds decreased, with epicatechin (52.71% of SF; 5.39 ± 0.45 µg/g DM) still being the dominant compound. It is known that a high intake of flavanols, which we found in fresh and fermented red cabbage, can be inversely related to the risk of cancer development [35].

In red beetroot without and after fermentation, we determined seventeen phenolic compounds (Table 4), including twelve phenolic acids and five flavonoids. Apart from six hydroxycinnamic acid derivatives (caffeic acid, chlorogenic acid, *p*-coumaric acid, ferulic acid, isoferulic acid, and sinapic acid) and five hydroxybenzoic acid derivatives (*m*-hydroxybenzoic acid, *p*-hydroxybenzoic acid, protocatechuic acid, syringic acid, and vanillic acid) that were determined in products of red beetroot, one phenylacetic acid derivative (*m*-hydroxyphenylacetic acid) was also detected. Furthermore, one flavanol (epicatechin), one flavone (orientin), and as many as three flavonols (kaempferol, quercetin, and rutin) were identified. A similar profile of phenolic compounds in fresh and fermented red cabbage was determined in the authors’ previous study [12]. Generally, it has been proved that the content of phenolic compounds in red beetroots subjected to fermentation processes has changed significantly (*p* < 0.05). This indicates that fermented foods are a good source of phenolic compounds compared to the unfermented ones.

The conducted analysis demonstrated that the total content of phenolic compounds in fresh red beetroot was 245.20 ± 8.40 µg/g DM, with phenolic acids constituting the main group (almost 90% of the SPF), and flavonoids being the minority (over 10% of the SPF). Moreover, phenolic compounds were found to occur in fresh red beetroot mainly in the bound forms (almost 76% of the SP), while free forms of phenolic acids accounted for only 22% of the SP. As has been mentioned above, red beetroots have a high content of phenolic acids (219.20 ± 7.60 µg/g DM). In detail, red beetroot was a significant source of hydroxybenzoic acid (>59% of the SP) and hydroxycinnamic acid derivatives (about 41% of the SP). When it comes to phenylacetic acid derivatives, beetroots were not an abundant source of these pigments (less than 0.1% of the SP). At the same time, our results indicate that phenolic acids occurred in the bound form mainly in red beetroot (approximately 76% of the SP), while their free form accounted for only 24% of the SP. For phenolic acids found in fresh red beetroot, syringic acid was the major compound (~45% of the SP), followed by isoferulic (~19% of the SP) and *p*-coumaric acids (~11% of the SP). Furthermore, the results clearly showed that fresh red beetroot was a considerable source of flavonoids. Flavanols (25.86 ± 0.80 µg/g DM) constituted the main group of flavonoids, with epicatechin being the major compound (over 99% of the SP). Flavonols accounted for less than 1% of the SF, and flavones (orientin) were not identified in fresh red beetroot. Interestingly, rutin was found only in fresh beetroot (this compound was not identified after the fermentation process). This is a potentially fascinating finding, since it is well known that rutin interacts synergistically with other bioactive substances [36]. It was also observed that flavonoids were mainly in the conjugated forms (99.46% of the SPF; 25.86 ± 0.80 µg/g DM), and their free forms accounted for only about 0.50% (0.14 ± 0.00 µg/g DM) of the SF. Our finding differs from those available in the literature [12]. This difference in the profile and content of phenolics compounds, as mentioned earlier, may result from varietal differences as well as the influence of biotic (plant diseases and pests) and abiotic (temperature, insolation, and precipitation) factors.

The fermentation process caused losses (degradation) of the content of phenolic compounds. The fermentation process led to a significant decrease in the sum of phenolic acids and flavonoids by about 40% (148.41 ± 6.26 µg/g DM; Table 3). In comparison to the fresh red beetroot, the reduction in phenolic acid and flavonoid content in fermented red beetroot was estimated to be 36.10% and 67.88%, respectively. In detail, our study showed that 32% and 39% of hydroxycinnamic and hydroxybenzoic acid derivatives, respectively, were degraded during the fermentation process. On the other hand, the content of phenylacetic acid derivatives in the fermented products was about 95% higher than that in the fresh red beetroot. A similar content of phenolic acids in fresh and fermented red cabbage products was determined in authors’ previous study [12]. However, it should be noted here that a significant reduction in phenolic compounds during food processing can be due to leaching [37]. Moreover, various factors, i.e., starters and enzyme activity, presence of oxygen, pH value, time, type, and temperature of fermentation, could also play a meaningful role and affect the nutritional quality of the final products [38]. In terms of the forms of phenolic acids in fermented red beetroot, our findings revealed that the process of spontaneous fermentation significantly affected the proportion of free and conjugated forms. On the other hand, the spontaneous fermentation caused an increase in the free forms of phenolic acid by about 34%. It was even more significant when it comes to conjugated forms, the proportions of which increased by more than 58%. One of the main factors influencing the phenolic compounds content is the starting microorganisms. Microorganisms have different abilities to produce enzymes and thus different abilities to break ester bonds. Hur et al. (2014) reported that the fermentation increased the content of soluble phenolic compounds due to the activity of glycosidases and lignin-degrading enzymes (cellulase, amylase, esterase, tannase, and glucosidase), leading to the liberation of phenolic compounds bound with the cell wall [39]. In the case of individual phenolic acids, it could be observed that the fermentation process increased the content of only *m*-hydroxyphenylacetic acid, while all other phenolic acids were degraded. Similarly, as in the case of the fresh red beetroot, syringic acid found in fermented products was the main compound from the phenolic acid group, and its contribution to the sum of phenolic compound was 71.45 ± 3.05 µg/g DM. In the case of flavonoids, the spontaneous fermentation of red beetroot led to a reduction in the sum of flavonoids (SF) by about 68% (8.35 ± 0.54 µg/g DM). The fermentation process decreased the content of all flavonols (~86%) and flavanols (~74%). Furthermore, flavones (orientin), which have not been identified in fresh beetroot, appeared in the fermented red beetroot, and their content was estimated at 1.68 ± 0.08 µg/g DM. Taking into account this information, it can be concluded that the reduction in phenolic compounds could be due to the oxidative enzymes that polymerize released phenolic compounds from cell walls [40]. Furthermore, the reduced phenolic compounds could be metabolized into other low molecular forms. This finding is in agreement with the observations made by Hunaefi et al. who demonstrate that lactic acid bacteria caused the degradation of dietary phenolics and, thereby, positively affected the final product, imparting it a nutritional composition, functionalities, distinctive aroma, taste, and texture [29]. As with phenolic acids, flavonoids were found in fermented red beetroot, mainly in the bound forms (approximately 80% of the SF). However, it is worth noting that the fermentation process caused a significant decrease in the content of these compounds, i.e., by about 75%. In contrast, the fermentation process increased the content of free forms of flavonoids by over 229%. Among the cellulolytic, pectinolytic, and lignolytic enzymes produced during the growth of microorganisms, β-glucosidase is described as the main enzyme responsible for catalyzing the hydrolysis of glycosidic bonds in alkyl and aryl-β-D-glucosides in order to release phenolic aglycone groups [38]. For flavonoids found in fermented red beetroot, epicatechin was the major compound (79.28% of the SF), as in the case of the fresh beetroot.

### 3.5. Anti-AGE Ability of Red Cabbage, Beetroot, and Their Products

**Figure 1:**
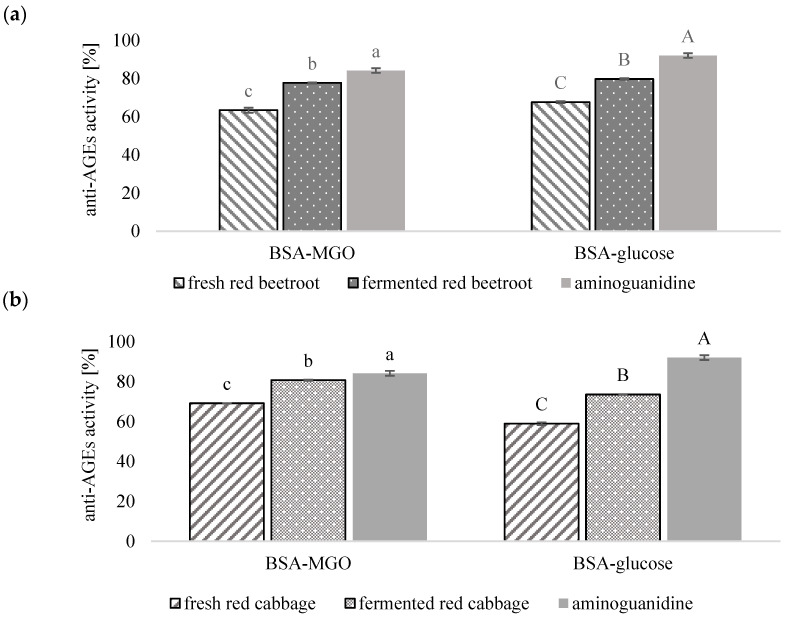
Anti-glycation properties of fresh and fermented red cabbage (**a**) fresh and (**b**) fermented red beetroot. Two model systems were of bovine serum albumin with methylglyoxal (BSA-MGO) and BSA with glucose (BSA–glucose). Bars followed by the same letter, calculated for each model system separately, were not significantly different (*p* < 0.05) according to Tukey’s test.

Figure 1 presents the results of determinations of the anti-glycation properties of red cabbage (fresh and fermented) and red beetroot (fresh and fermented). These properties were determined *in vitro* in two model systems: BSA-MGO and BSA–glucose. These systems are deployed to evaluate the inhibitory effects on protein glycation induced by MGO (a potent precursor of AGEs) and glucose (reduced sugar, the high content of which is detected in the blood of patients with diabetes). Aminoguanidine (AG) was used as a positive control, and the degrees of glycation were analyzed for the development of specific fluorescence. The AG ensured 84.13% (BSA-MGO) and 92.00% (BSA–glucose) inhibition of AGE formation.

The anti-AGE ability of fresh red cabbage extracts was determined in the BSA-MGO model to be 69.13%. In the BSA–glucose model, the analyzed extracts of red cabbage exhibited a higher AGE inhibitory status than that of red cabbage reported by Thilavech et al. [41]. The red cabbage extracts had a similar ability to trap MGO compared to extracts of another *Brassica* vegetable—cauliflower—at a concentration of 0.5 mg/mL [41]. Furthermore, almost 17% and 25% increases in the anti-glycation activity were determined in BSA-MGO and BSA–glucose systems for the fermented red cabbage, respectively. The anti-glycation properties of the fermented red cabbage extracts were only about 4% and 8% lower compared to the reference material, i.e., AG. A significant increase, from 63.37% to 77.64% (~23% increase), was also noticed in the anti-AGE ability of fermented red beetroot in the BSA-MGO model and an increase from 67.61% to 79.73% (~18%) in the BSA–glucose model. The anti-AGE ability of fermented red beetroot was lower compared to aminoguanidine’s ability only by ~8% (BSA-MGO) and ~13% (BSA–glucose). The results obtained using the BSA-MGO model were strongly correlated (r = 0.850) with those found using the BSA–glucose model system, which was in line with the findings reported by Starowicz and Zieliński [6]. Moreover, the anti-AGEs abilities of fermented vegetables tested were similar to those obtained for clove, star anise, and allspice [6]. Hence, it may be concluded that the fermentation process induced the anti-glycation features of vegetables. Our findings are in agreement with observations made by Kuda et al., who noticed that fermentation of algae increased their activity against AGE formation [9]. The elevated anti-AGE ability might be related to an increased content of phenolics in the fermented products [30]. A few different mechanisms of the anti-glycation effects of phenolics are described [42]. Phenolic acids and flavonoids are potent inhibitors of the formation of MGO and/or detrimental AGEs as well as reactive carbonyl species [42]. Therefore, the total contents of phenolics (TPC) and flavonoids (TF) of fresh and processed vegetables were determined to find a relationship with their anti-glycation abilities.

### 3.6. Correlation Studies

In order to estimate the relationship between the content of bioactive compounds and the antioxidant activity in red cabbage products, Pearson’s correlation coefficients were calculated. In the fresh and the fermented red cabbage, a strong correlation was found between antioxidant capacity and TPC (r = 0.959), TFC (r = 0.922), and anthocyanin contents (r = 0.987). The results of the present study are in agreement with findings reported by Hounsome et al., which confirmed a strong correlation between antioxidant activity and the total phenolic content in *Brassica* species [43]. It seems that the contents of the identified bioactive compounds increased mostly in the fermentation of red cabbage. Moreover, results of BSA-MGO vs. BSA–glucose models (r = 0.850), BSA-MGO/glucose vs. SP, BSA-MGO/glucose vs. SPF, BSA-MGO/glucose vs. TPC, and BSA-MGO/glucose vs. TFC (r = 1.000) were positively correlated in the case of red cabbage products. Conversely, in our study, only SF was negatively correlated with the results of anti-AGE ability measured using these two models. This probably stems from the fact that only flavonoid content has no influence on the anti-AGE properties of fermented red cabbage.

## 4. Conclusions

As already mentioned, this is the first study that shows changes in the bioactive compounds (phenolic acids, flavonoids, anthocyanins, and betalains) and anti-glycation properties during the spontaneous fermentation of red beetroot and red cabbage. The conclusion of this study shows that the fermentation process of red cabbage and beetroot is a suitable tool to increase the content of phenolic compounds, and therefore, it might be related to their enhanced functional properties, e.g., the significant inhibitory effect against AGE formation/accumulation by these products. On the other hand, the application of the fermentation process decreased the amount of red pigments. In the case of the sum of anthocyanins, about 8% degradation was observed, whereas betalain content was reduced by six times after fermentation. It means that the usage of microorganisms should be implemented in more controlled conditions. Moreover, the data that we obtained on the complete profile of phenolic acids, flavonoids, anthocyanins, and/or betalains of red beetroot and red cabbage are important as they can help increase the functional properties of these compounds in the final processed vegetable products. The potential health benefits of beetroot and its fermented products as natural AGE inhibitors make them valuable additions to a balanced and health-conscious diet. Incorporating these chosen vegetables into our meals may not only add a burst of color and flavor but also offer a natural way to support our body’s defenses against AGEs and promote overall well-being. Future research should focus on the influence of industrial processing on biologically active compounds of red cabbage and red beetroot products and their bioavailability after consumption of the final products. Therefore, when planning vegetable processing, fermentation should be taken into account as a promising tool to modulate pro-healthy properties of the final products.

## Figures and Tables

**Table 1:** Content of anthocyanins in fresh and fermented red cabbage.

No	Abbreviation	Compounds	Red Cabbage	
			Fresh	Fermented
			[µg/g DM]	
1	Cy3diG5G	cyanidin 3-diglucoside-5-glucoside	1.84 ± 0.18 ^a^	1.91 ± 0.08 ^a^
2	Cy3G5G	cyanidin 3-glucoside-5-glucoside	0.29 ± 0.00 ^a^	0.19 ± 0.00 ^b^
3	Cy3(sin)diG5G	cyanidin 3-(sinapoyl)-diglucoside-5-glucoside	0.94 ± 0.01 ^a^	0.90 ± 0.05 ^a^
4	Cy3(sin)triG5G	cyanidin 3-(sinapoyl)-triglucoside-5-glucoside	0.38 ± 0.05 ^a^	0.36 ± 0.04 ^a^
5	Cy3(caf)(*p*-cum)diG5G	cyanidin 3-(caffeoyl)(*p*-coumaroyl)-diglucoside-5-glucoside	0.42 ± 0.02 ^a^	0.29 ± 0.02 ^b^
6	Cy3(fer)triG5G	cyanidin 3-(feruloyl)-triglucoside-5-glucoside	0.28 ± 0.02 ^a^	0.20 ± 0.01 ^b^
7	Cy3(sin)triG5G	cyanidin 3- (sinapoyl)-triglucoside-5-glucoside	0.15 ± 0.00 ^a^	0.11 ± 0.00 ^b^
8	Cy3(fer)(fer)triG5G	cyanidin 3-(feruloyl)(feruloyl)-triglucoside-5-glucoside	0.45 ± 0.01 ^a^	0.27 ± 0.01 ^b^
9	Cy3(fer)diG5G	cyanidin 3-(feruloyl)-diglucoside-5-glucoside	0.48 ± 0.01 ^a^	0.34 ± 0.01 ^b^
10	Cy3(fer)(sin)triG5G	cyanidin 3-(feruloyl)(sinapoyl)-triglucoside-5-glucoside	0.17 ± 0.01 ^a^	0.14 ± 0.01 ^a^
11	Cy3(*p*-cum)diG5G	cyanidin 3-(*p*-coumaroyl)-diglucoside-5-glucoside;	0.70 ± 0.02 ^b^	0.85 ± 0.02 ^a^
12	Cy3(caf)(*p*-cum)diG5G	cyanidin 3-(caffeoyl)(*p*-coumaroyl)-diglucoside-5-glucoside	0.07 ± 0.00	nd
13	Cy3(fer)diG5G	cyanidin 3-(feruloyl)-diglucoside-5-glucoside	0.44 ± 0.02 ^b^	0.63 ± 0.01 ^a^
14	Cy3(sin)diG5G	cyanidin 3-(sinapoyl)-diglucoside-5-glucoside	0.87 ± 0.01 ^b^	0.91 ± 0.01 ^a^
15	Cy3(fer)G5G	cyanidin 3-(feruloyl)-glucoside-5-glucoside	0.12 ± 0.00 ^a^	0.10 ± 0.00 ^b^
16	Cy3(sin)G5G	cyanidin 3-(sinapoyl)-glucoside-5-glucoside	0.14 ± 0.01 ^a^	0.11 ± 0.01 ^a^
17	Cy3(fer)(fer)diG5G	cyanidin 3-(feruloyl)(feruloyl)-diglucoside-5-glucoside	1.10 ± 0.01 ^a^	0.91 ± 0.02 ^b^
18	Cy3(fer)(sin)diG5G	cyanidin 3-(feruloyl)(sinapoyl)-diglucoside-5-glucoside	1.13 ± 0.00 ^a^	0.98 ± 0.01 ^b^
19	Cy3(sin)(sin)diG5 G	cyanidin 3-(sinapoyl)(sinapoyl)-diglucoside-5-glucoside	2.84 ± 0.03 ^a^	2.61 ± 0.03 ^b^
**Sum of anthocyanins**			**12.81 ± 0.41 ^a^**	**11.81 ± 0.34 ^b^**

Abbreviations: nd—not detected; DM—dry matter. Mean results ± SD, followed by the same letter in the row, calculated for each compound separately (^a^, ^b^) were not significantly different (*p* < 0.05) according to Tukey’s test.

**Table 2:** Content of betalains in fresh red beetroot and its fermented product.

No	Compound	Red Beetroot	
		Fresh	Fermented
		[µg/g DM]	
1	betanin	2.54 ± 0.13 ^a^	0.02 ± 0.00 ^b^
2	isobetanin	0.77 ± 0.02 ^a^	0.00 ± 0.00 ^b^
3	17-decarboxy-betanin	0.06 ± 0.00 ^a^	0.06 ± 0.00 ^a^
4	2,17-bidecarboxy-betanin	0.66 ± 0.01 ^a^	0.34 ± 0.01 ^b^
5	2,15,17-tridecarboxy-betanin	1.09 ± 0.03 ^a^	0.23 ± 0.01 ^b^
6	vulgaxantin I	0.17 ± 0.00 ^a^	0.04 ± 0.00 ^b^
7	vulgaxantin II	0.46 ± 0.01 ^a^	0.25 ± 0.01 ^b^
**Total betalains content**		**5.75 ± 0.20 ^a^**	**0.94 ± 0.03 ^b^**

Abbreviations: DM, dry matter. Mean results ± SD followed by the same letter in the row, calculated for each compound separately (^a^, ^b^), were not significantly different (*p* < 0.05) according to Tukey’s test.

**Table 3:** Contents of phenolic acids and flavonoids and total phenolic and flavonoid contents determined in red cabbage (fresh and fermented).

No	Compound	Red Cabbage					
		Fresh			Fermented		
		F	C	F + C	F	C	F + C
***Phenolic acids [µg*** **/*g DM]***							
1	caffeic acid	1.45 ± 0.14	46.84 ± 2.38	48.29 ± 2.38 ^b^	1.12 ± 0.10	61.52 ± 1.37	62.63 ± 1.46 ^a^
2	chlorogenic acid	0.06 ± 0.01	0.03 ± 0.00	0.09 ± 0.00 ^a^	0.05 ± 0.00	0.01 ± 0.00	0.06 ± 0.00 ^b^
3	*p*-coumaric acid	6.61 ± 0.23	27.32 ± 2.30	33.93 ± 2.30 ^b^	0.91 ± 0.10	28.16 ± 1.14	29.07 ± 1.23 ^a^
4	ferulic acid	0.70 ± 0.00	4.19 ± 0.16	4.89 ± 0.16 ^b^	1.71 ± 0.10	4.29 ± 0.11	6.00 ± 0.21 ^b^
5	*p*-hydroxybenzoic acid	1.35 ± 0.04	16.16 ± 0.53	17.50 ± 0.53 ^b^	13.14 ± 0.39	15.77 ± 0.46	28.91 ± 0.85 ^a^
6	*m*-hydroxyphenylacetic acid	0.03 ± 0.00	11.99 ± 0.40	12.02 ± 0.40 ^b^	0.41 ± 0.02	14.23 ± 0.45	14.64 ± 0.47 ^a^
7	*m*-hydroxybenzoic acid	1.23 ± 0.01	2.50 ± 0.18	3.73 ± 0.18 ^a^	1.35 ± 0.07	2.88 ± 0.20	4.22 ± 0.26 ^a^
8	isoferulic acid	3.05 ± 0.02	33.62 ± 2.54	36.68 ± 2.54 ^b^	3.18 ± 0.16	40.49 ± 2.06	43.67 ± 2.22 ^a^
9	protocatechuic acid	nd	1.99 ± 0.09	1.99 ± 0.09 ^b^	nd	3.32 ± 0.21	3.32 ± 0.21 ^a^
10	sinapic acid	27.63 ± 1.67	50.80 ± 3.84	78.43 ± 3.84 ^b^	45.89 ± 1.56	86.85 ± 3.40	132.75 ± 4.96 ^a^
11	syringic acid	15.02 ± 0.20	24.61 ± 0.46	39.63 ± 0.46 ^a^	19.89 ± 0.81	24.39 ± 2.03	44.27 ± 2.83 ^a^
12	vanillic acid	0.51 ± 0.06	23.97 ± 0.49	24.47 ± 0.49 ^a^	3.34 ± 0.05	13.07 ± 0.12	16.40 ± 0.16 ^b^
	**Sum of phenolic acids (SP)**	**57.63 ± 2.37**	**244.01 ± 13.37**	**301.64 ± 13.37 ^b^**	**90.99 ± 3.34**	**294.97 ± 11.54**	**385.95 ± 14.88 ^a^**
***Flavonoids [µg*** **/*g DM]***							
13	apigenin	0.95 ± 0.09	0.00 ± 0.00	0.95 ± 0.00 ^a^	0.00 ± 0.00	0.00 ± 0.00	0.00 ± 0.00 ^b^
14	epicatechin	nd	19.81 ± 0.45	19.81 ± 0.45 ^a^	nd	5.39 ± 0.45	5.39 ± 0.45 ^b^
15	kaempferol	0.16 ± 0.01	0.54 ± 0.04	0.70 ± 0.04 ^b^	0.27 ± 0.01	0.52 ± 0.02	0.79 ± 0.03 ^a^
16	orientin	nd	2.36 ± 0.13	2.36 ± 0.13 ^a^	nd	2.71 ± 0.28	2.71 ± 0.28 ^a^
17	quercetin	0.04 ± 0.00	0.32 ± 0.01	0.36 ± 0.01 ^b^	0.30 ± 0.01	0.17 ± 0.02	0.47 ± 0.03 ^a^
18	rutin	0.05 ± 0.00	nd	0.05 ± 0.00 ^b^	0.82 ± 0.08	nd	0.82 ± 0.08 ^a^
19	vitexin	nd	0.05 ± 0.00	0.05 ± 0.00 ^a^	0.01 ± 0.00	0.04 ± 0.00	0.04 ± 0.00 ^a^
	**Sum of flavonoids (SF)**	**1.20 ± 0.10**	**23.09 ± 0.64**	**24.27 ± 0.64 ^a^**	**1.40 ± 0.11**	**8.82 ± 0.76**	**10.22 ± 0.87 ^b^**
	**Sum of phenolic acids and flavonoids (SPF)**	**58.83 ± 2.47**	**267.1 ± 14.01**	**325.91 ± 14.01 ^b^**	**92.39 ± 3.45**	**303.79 ± 12.30**	**396.17 ± 15.75 ^a^**
	**TPC [mg GA/g DM]**	**36.49 ± 1.25 ^b^**			**40.14 ± 0.98 ^a^**		
	**TFC [mg Q/g DM]**	**0.72 ± 0.03 ^b^**			**0.82 ± 0.02 ^a^**		

Abbreviations: F, free forms of phenolic acids or flavonoids; C, conjugated forms of phenolic acids or flavonoids released from soluble esters and glycosides; nd, not detected; GA, gallic acid; Q, quercetin; DM, dry matter; TPC, total phenolic content; and TFC, total flavonoid content. Mean results ± SD followed by the same letter, calculated for each compound separately, were not significantly different (*p* < 0.05) according to Tukey’s test.

**Table 4:** Contents of phenolic acids and flavonoids and total phenolic and flavonoid contents in fresh red beetroot and its fermented products.

No	Compound	Fresh			Fermented		
		F	C	F + C	F	C	F + C
*Phenolic acids [µg*/*g DM]*							
1	caffeic acid	0.20 ± 0.03	3.94 ± 0.02	4.14 ± 0.04 ^a^	0.98 ± 0.07	2.90 ± 0.15	3.88 ± 0.21 ^a^
2	chlorogenic acid	nd	0.02 ± 0.00	0.02 ± 0.00 ^a^	nd	0.01 ± 0.00	0.01 ± 0.00 ^b^
3	*p*-coumaric acid	0.21 ± 0.01	24.86 ± 0.15	25.07 ± 0.16 ^a^	5.84 ± 0.04	15.91 ± 0.60	21.75 ± 0.64 ^b^
4	ferulic acid	3.38 ± 0.11	4.42 ± 0.38	7.79 ± 0.48 ^a^	4.19 ± 0.24	2.71 ± 0.26	6.90 ± 0.50 ^a^
5	*m*-hydroxybenzoic acid	0.60 ± 0.00	1.00 ± 0.06	1.60 ± 0.07 ^a^	0.86 ± 0.02	0.49 ± 0.02	1.34 ± 0.04 ^b^
6	*p*-hydroxybenzoic acid	1.30 ± 0.03	15.57 ± 0.34	16.86 ± 0.37 ^a^	2.72 ± 0.05	2.72 ± 0.05	5.43 ± 0.09 ^b^
7	*m*-hydroxyphenylacetic acid	0.02 ± 0.00	0.19 ± 0.00	0.21 ± 0.01 ^b^	0.02 ± 0.00	0.38 ± 0.02	0.41 ± 0.02 ^a^
8	isoferulic acid	6.90 ± 0.23	35.10 ± 0.28	42.00 ± 0.51 ^a^	10.45 ± 0.66	15.56 ± 0.38	26.01 ± 1.04 ^b^
9	protocatechuic acid	0.02 ± 0.00	0.33 ± 0.01	0.35 ± 0.01 ^a^	0.01 ± 0.00	0.09 ± 0.00	0.09 ± 0.00 ^b^
10	sinapic acid	1.69 ± 0.06	8.59 ± 0.37	10.28 ± 0.43 ^a^	0.43 ± 0.00	1.81 ± 0.10	2.23 ± 0.10 ^b^
11	syringic acid	38.44 ± 2.22	59.33 ± 2.72	97.77 ± 4.94 ^a^	45.32 ± 1.94	26.13 ± 1.11	71.45 ± 3.05 ^b^
12	vanillic acid	0.20 ± 0.01	12.91 ± 0.57	13.11 ± 0.58 ^a^	nd	0.56 ± 0.03	0.56 ± 0.03 ^b^
**Sum of phenolic acids (SP)**		**52.96 ± 2.70**	**166.26 ± 4.90**	**219.20 ± 7.60 ^a^**	**70.82 ± 3.02**	**69.27 ± 2.72**	**140.06 ± 5.72 ^b^**
*Flavonoids [µg*/*g DM]*							
13	epicatechin	nd	25.82 ± 0.79	25.82 ± 0.79 ^a^	nd	6.62 ± 0.46	6.62 ± 0.46 ^b^
14	kaempferol	0.06 ± 0.00	nd	0.06 ± 0.00 ^a^	0.04 ± 0.00	nd	0.04 ± 0.00 ^b^
15	orientin	nd	nd	nd	1.68 ± 0.08	nd	1.68 ± 0.08 ^a^
16	quercetin	0.03 ± 0.00	0.04 ± 0.01	0.07 ± 0.01 ^a^	0.00 ± 0.00	0.00 ± 0.00	0.01 ± 0.00 ^b^
17	rutin	0.05 ± 0.00	nd	0.05 ± 0.00 ^a^	nd	nd	nd
**Sum of flavonoids (SF)**		**0.14 ± 0.00**	**25.86 ± 0.80**	**26.00 ± 0.80 ^a^**	**1.72 ± 0.08**	**6.62 ± 0.46**	**8.35 ± 0.54 ^b^**
**TPC [mg GA/g DM]**		**33.73 ± 0.79 ^a^**			**26.36 ± 1.63 ^b^**		
**TFC [mg Q/g DM]**		**2.11 ± 0.03 ^a^**			**1.45 ± 0.03 ^b^**		

Abbreviations: F, free forms of phenolic acids or flavonoids; C, conjugated forms of phenolic acids or flavonoids released from soluble esters and glycosides; nd, not detected; GA, gallic acid; Q, quercetin; DM, dry matter; TPC, total phenolic content; and TFC, total flavonoid content. Mean results ± SD followed by the same letter, calculated for each compound separately, were not significantly different (*p* < 0.05) according to Tukey’s test.

## Data Availability

The original contributions presented in the study are included in the article/Supplementary Materials, further inquiries can be directed to the corresponding author.

## Supplementary Materials

The following supporting information can be downloaded at: https://www.mdpi.com/article/10.3390/foods13121791/s1, Table S1: The UV–Vis and MS data of red cabbage anthocyanins; Table S2: The MS data of red beetroot betalains; Table S3: The MS data of red cabbage and red beetroot phenolic acids and flavonoids.
