# Supplementary material for: Unraveling the In Vitro Anti-Advanced Glycation End-Product (Anti-AGE) Potential of Fermented Red Cabbage and Beetroot: Insights into Composition and Activities
Source: Foods. 2024 Jun 7;13(12):1791. doi: 10.3390/foods13121791 (PMC11203313; doi:10.3390/foods13121791)
Supplement: Supplementary file 1 [file foods-13-01791-s001.zip › foods-3011736-supplementary.pdf]

Table S1: The UV–Vis and MS data of red cabbage anthocyanins.

No	Abbreviation	Compounds	λ_{vis} (nm)	λ_{acyl} (nm)	$[M]^+$ (*m/z*)	MS/MS (*m/z*)	R_t (min)	MS/MS product ion spectrum
1	Cy3diG5G	cyanidin 3-diglucoside-5-glucoside	513	x	773	611/449/287	1.82	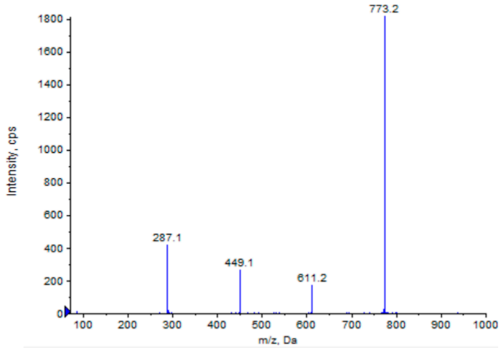
2	Cy3G5G	cyanidin 3-glucoside-5-glucoside	512	x	611	449/287	1.85	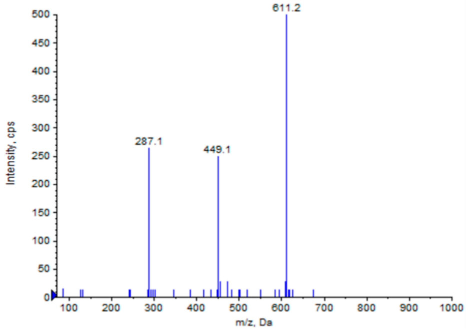
3	Cy3(sin)diG5G	cyanidin 3-(sinapoyl)-diglucoside-5-glucoside	527	330	979	817/449/287	1.93	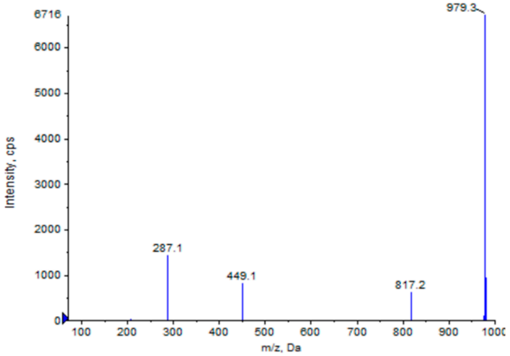

4	Cy3(sin)triG5G	cyanidin 3-(sinapoyl)-triglucoside-5-glucoside	525	321	1141	979/449/287	1.94	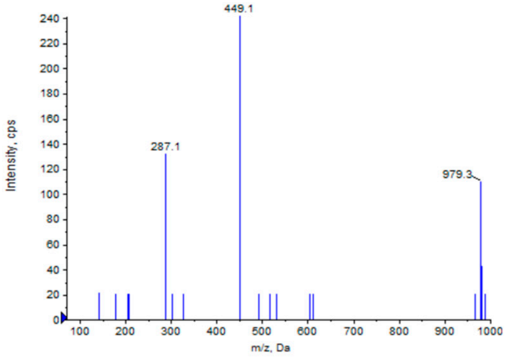
5	Cy3(caf)(*p*-cum)diG5G	cyanidin 3-(caffeoyl)(*p*-coumaroyl)-diglucoside-5-glucoside	521	314	1081	919/449/287	1.95	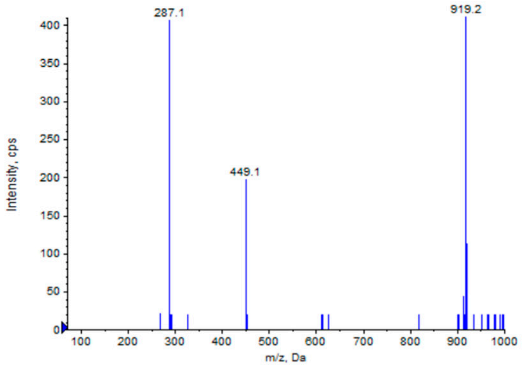
6	Cy3(fer)triG5G	cyanidin 3-(feruloyl)-triglucoside-5-glucoside	522	320	1111	949/449/287	1.96	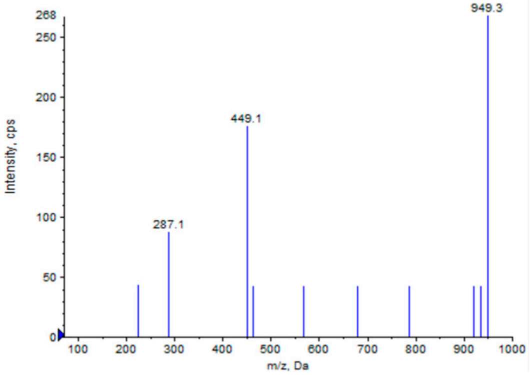

7	Cy3(sin)triG5G	cyanidin 3- (sinapoyl)-triglucoside-5-glucoside	525	321	1141	979/449/287	1.97	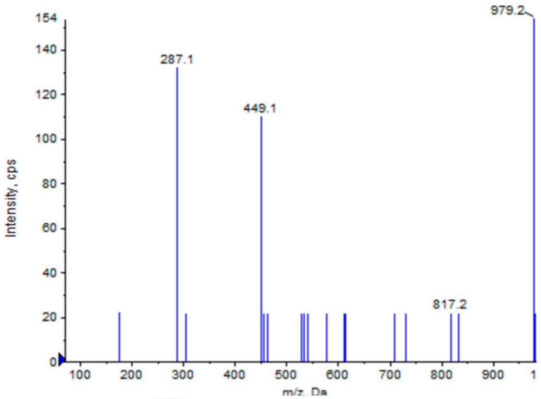
8	Cy3(fer)(fer)triG5G	cyanidin 3-(feruloyl)(feruloyl)-triglucoside-5-glucoside	536	321	1287	1125/449/287	1.98	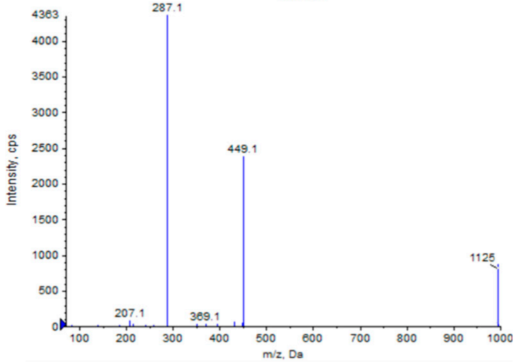
9	Cy3(fer)diG5G	cyanidin 3-(feruloyl)-diglucoside-5-glucoside	522	328	949	787/449/287	2.00	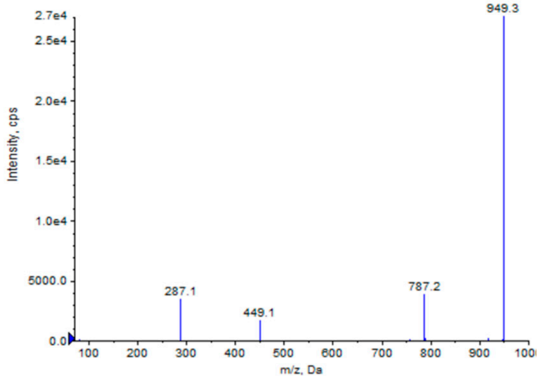

10	Cy3(fer)(sin)triG5G	cyanidin 3-(feruloyl)(sinapoyl)-triglucoside-5-glucoside	536	324	1317	1155/449/287	2.01	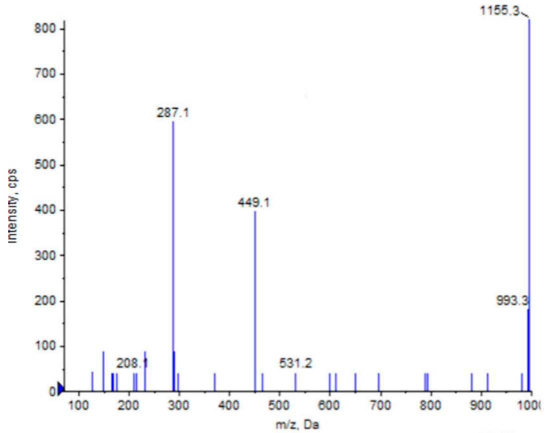
11	Cy3(*p*-cum)diG5G	cyanidin 3-(*p*-coumaroyl)-diglucoside-5-glucoside	521	312	919	757/449/287	2.08	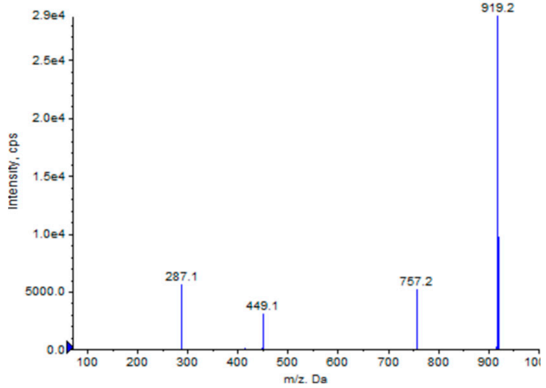
12	Cy3(caf)(*p*-cum)diG5G	cyanidin 3-(caffeoyl)(*p*-coumaroyl)-diglucoside-5-glucoside	521	314	1081	919/449/287	2.09	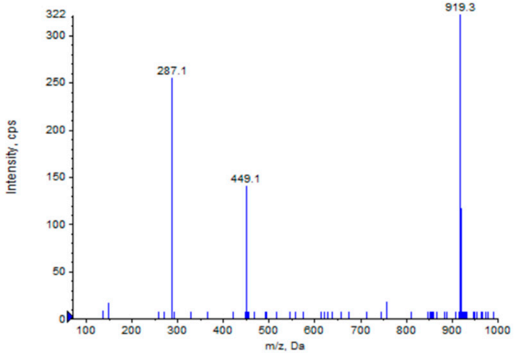

13	Cy3(fer)diG5G	cyanidin 3-(feruloyl)-diglucoside-5-glucoside	523	329	949	787/449/287	2.10	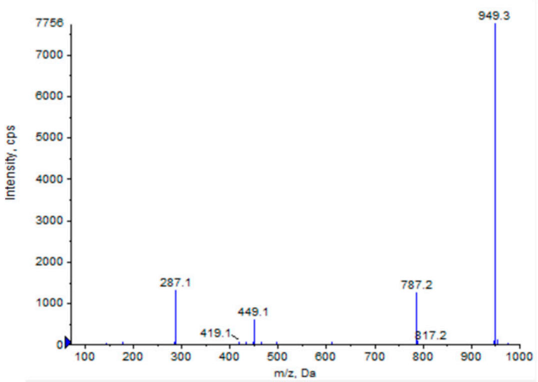
14	Cy3(sin)diG5G	cyanidin 3-(sinapoyl)-diglucoside-5-glucoside	526	328	979	817/449/287	2.12	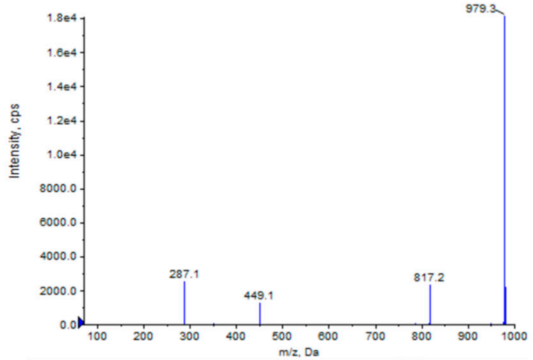
15	Cy3(fer)G5G	cyanidin 3-(feruloyl)-glucoside-5-glucoside	522	328	787	449/287	2.13	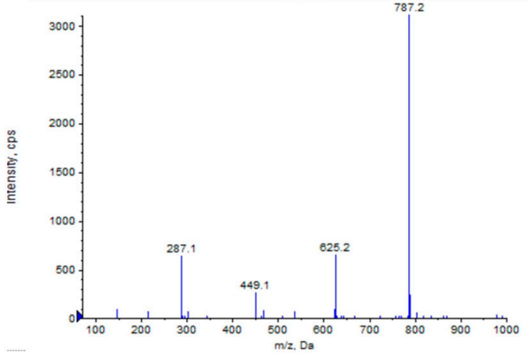

16	Cy3(sin)G5G	cyanidin 3-(sinapoyl)-glucoside-5-glucoside	527	330	817	449/287	2.14	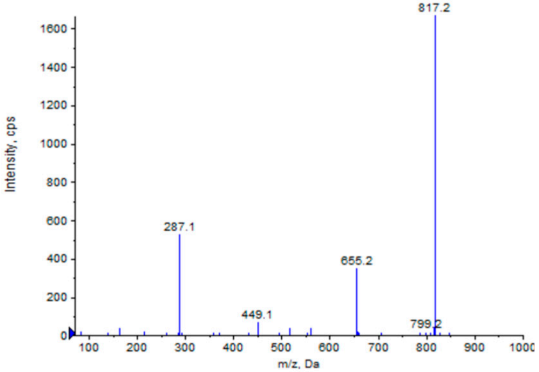
17	Cy3(fer)(fer)diG5G	cyanidin 3-(feruloyl)(feruloyl)-diglucoside-5-glucoside	535	328	1125	963/449/287	2.15	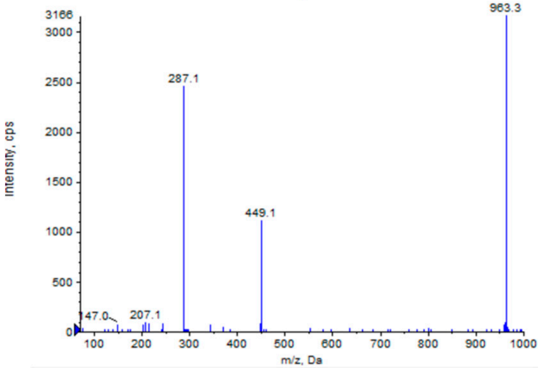
18	Cy3(fer)(sin)diG5G	cyanidin 3-(feruloyl)(sinapoyl)-diglucoside-5-glucoside	535	330	1155	993/449/287	2.16	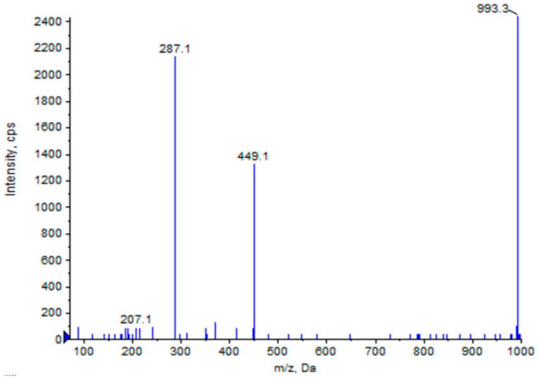

19	Cy3(sin)(sin)diG5G	cyanidin 3-(sinapoyl)(sinapoyl)-diglucoside-5-glucoside	535	332	1185	1023/449/287	2.18	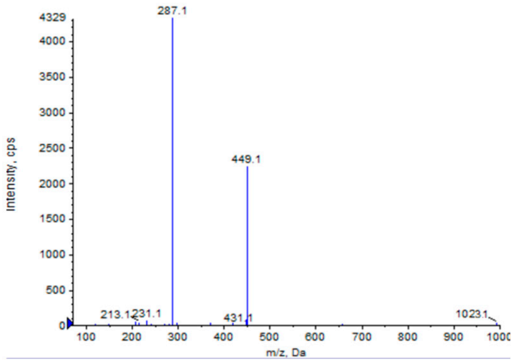

Table S2: The MS data of red beetroot betalains.

No	Compound	$[M]^+$ (m/z)	MS/MS (*m/z*)	Retention time [min]	MS/MS product ion spectrum
			Betacyanins		
**1**	Betanin	551.1	389.1	1.29	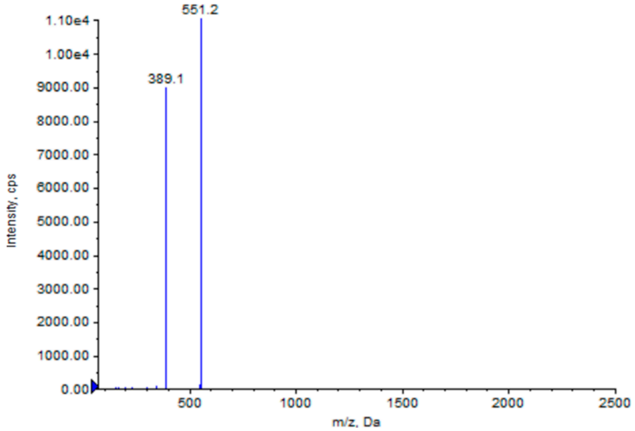
**2**	Isobetanin	551.1	389.1	1.34	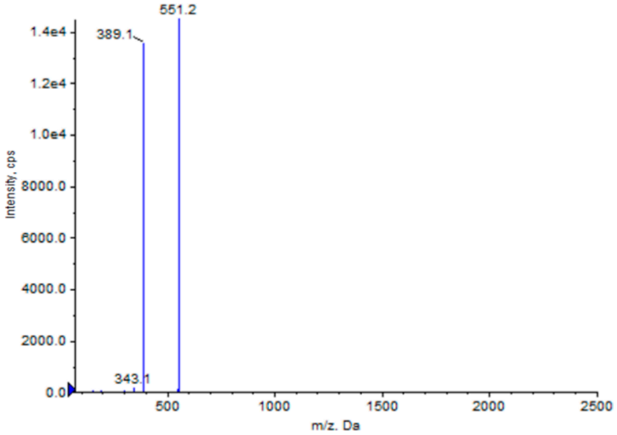

**3**	17-Decarboxy-betanin	507.1	345.0	1.39	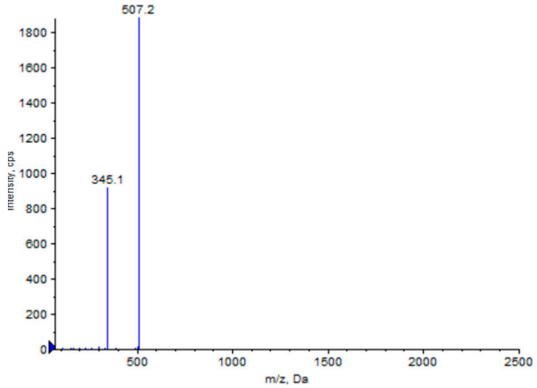
**4**	2,17-Bidecarboxy-betanin	463.1	301.1	1.56	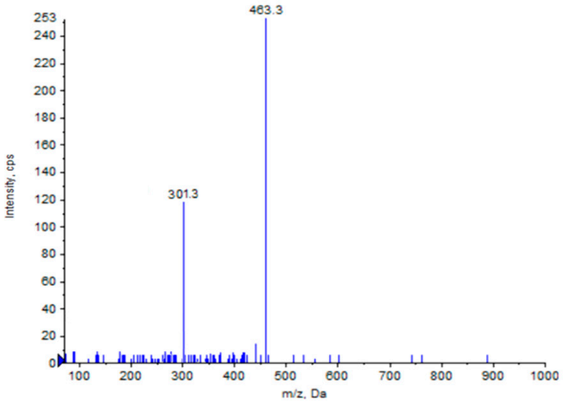

**5**	2,15,17-Tridecarboxy-betanin	419.1	257.1	2.02	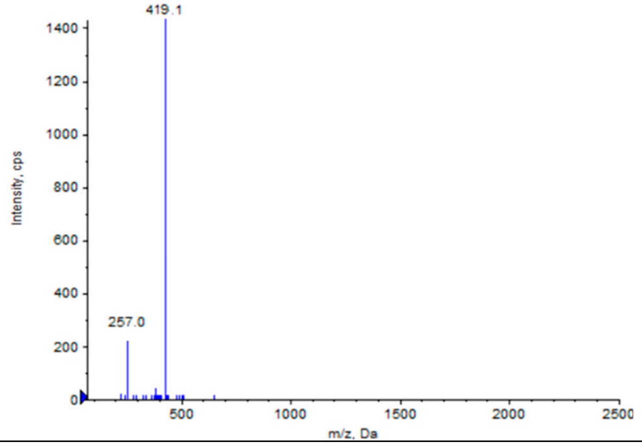
		Betaxanthins			
**6**	Vulgaxanthin I	340.1	323.1/277.1	1.01	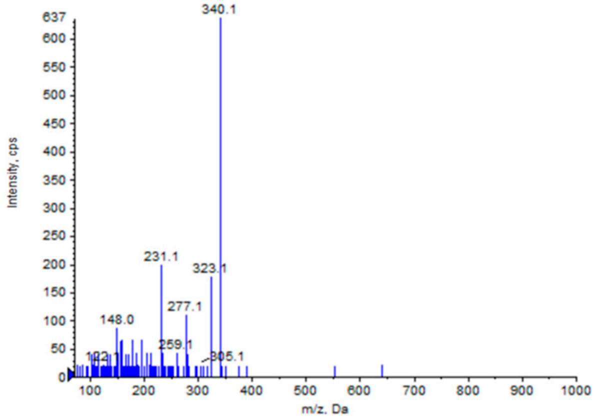

**7**	Vulgaxantin II	341.1	297.1	1.12	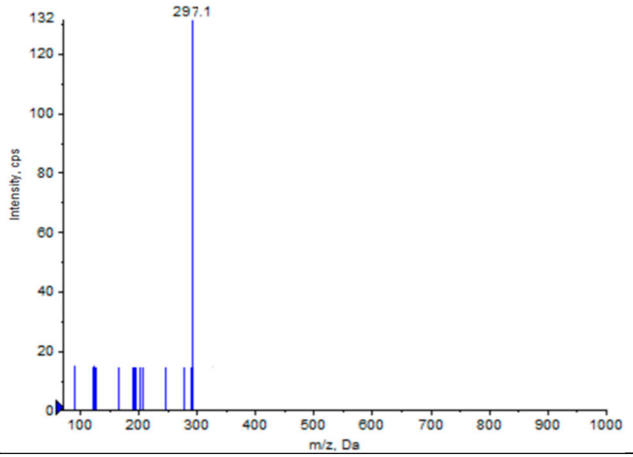

Table S3: The MS data of red cabbage and red beetroot phenolic acids and flavonoids.

No	Compounds	R_t (min)	$[M]^-$ (m/z)	MS/MS (m/z)	MS/MS product ion spectrum
			Phenolic acids		
**1**	Caffeic acid	2.41	179	135/107	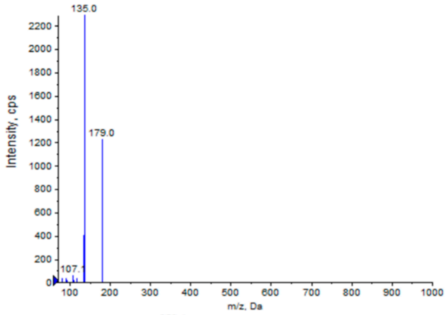
**2**	Chlorogenic acid	2.21	353	191/179/147	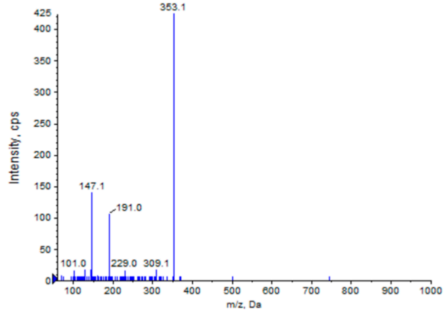
**3**	*p*-Coumaric acid	2.51	163	119/93	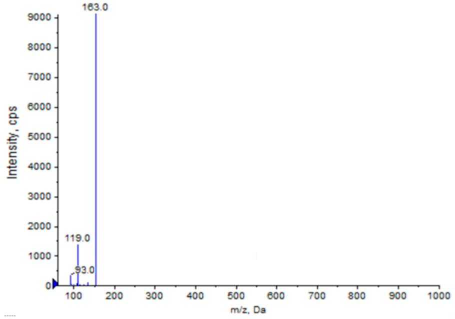

**4**	Ferulic acid	2.52	193	178/134	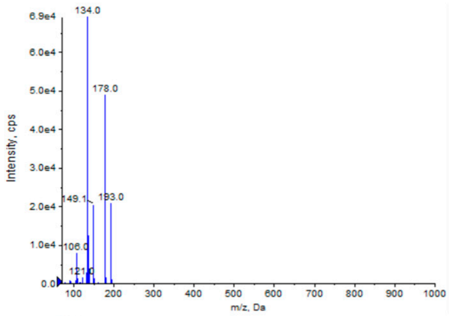
**5**	*p*-Hydroxybenzoic acid	2.30	137	93/65	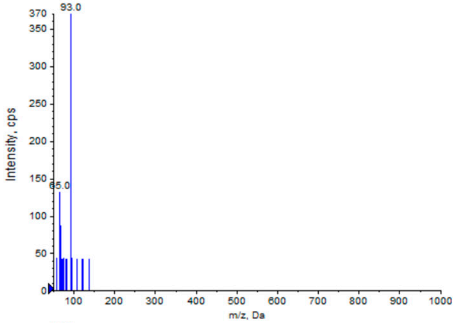
**6**	*m*-Hydroxyphenylacetic acid	2.41	151	135/107/93	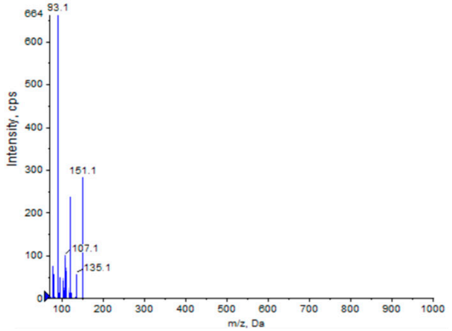

**7**	*m*-Hydroxybenzoic acid	2.41	137	93/65	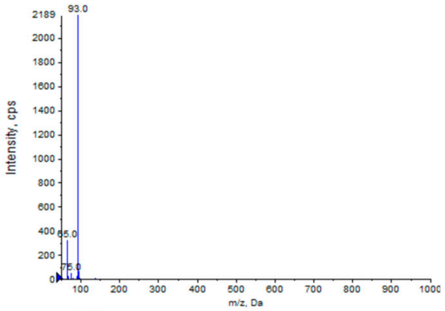
**8**	Isoferulic acid	2.58	193	178/134	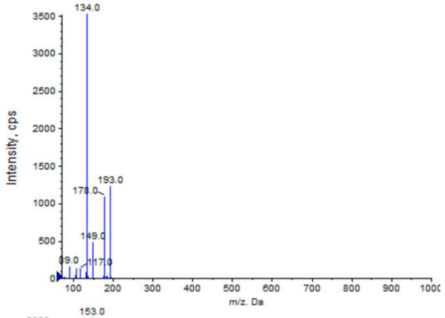
**9**	Protocatechuic acid	1.91	153	91/81	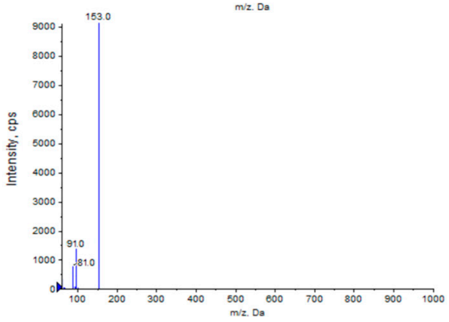

**10**	Sinapic acid	2.55	223	208/179/164	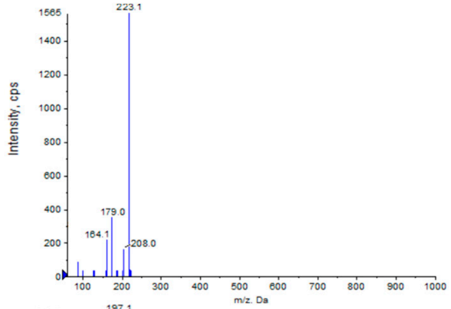
**11**	Syringic acid	2.45	197	182/153	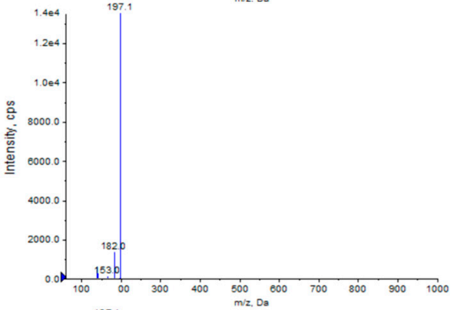
**12**	Vanillic acid	2.27	167	123/108/91	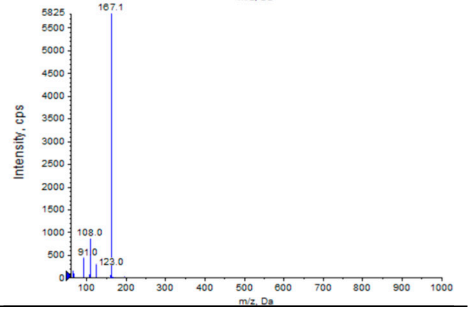

**13**	Apigenin	2.82	269	225/151/117	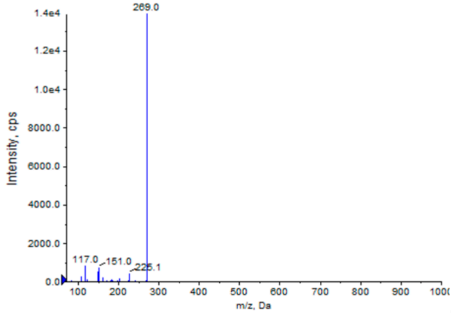
**14**	Epicatechin	2.33	289	245/203/109	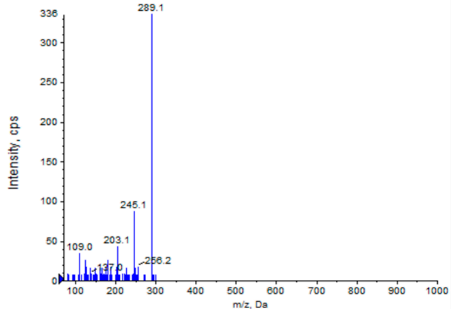
**15**	Kaempferol	2.92	285	185/93	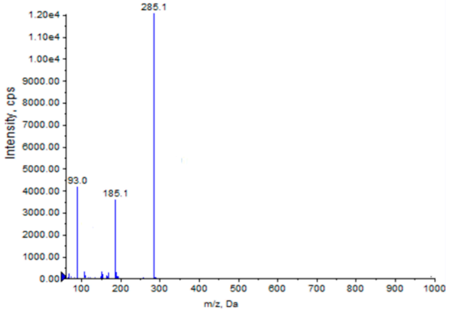

**16**	Orientin	3.00	447	357/339/296	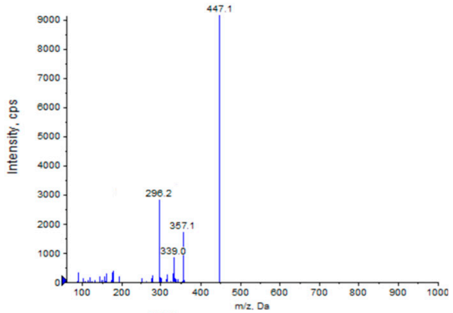
**17**	Quercetin	2.72	301	179/151	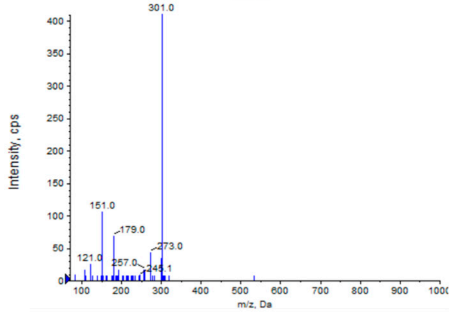
**18**	Rutin	2.34	609	463/301	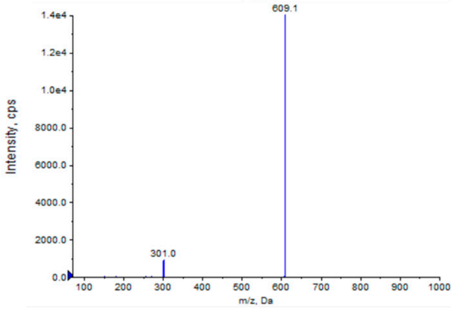

**19**	Vitexin	1.92	431	323/311/283	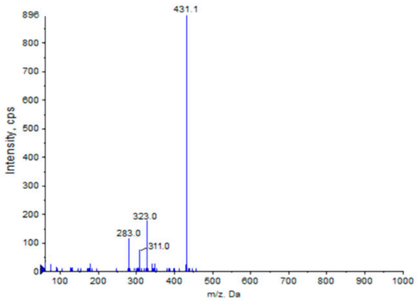
